# Comparative Neoplasm Risk of Mirabegron and Antimuscarinic Therapy in Overactive Bladder: A Multicenter Target Trial Emulation

**DOI:** 10.3390/cancers18203279

**Published:** 2026-10-12

**Authors:** Fu-Shun Yen, James Cheng-Chung Wei, Chih-Cheng Hsu, Chii-Min Hwu, Yao-Min Hung, Renin Chang, Tzu-Ping Lin

**Affiliations:** 1 Dr. Yen’s Clinic, No. 15, Shanying Road, Gueishan District, Taoyuan 33354, Taiwan; yenfushun@gmail.com; 2 Institute of Medicine, Chung Shan Medical University, Taichung 40201, Taiwan; 3 Department of Allergy, Immunology & Rheumatology, Chung Shan Medical University Hospital, No. 110, Sec. 1, Jianguo N. Rd., South District, Taichung 40201, Taiwan; 4 Graduate Institute of Integrated Medicine, China Medical University, No. 91, Hsueh-Shih Road, Taichung 40402, Taiwan; 5 Institute of Population Health Sciences, National Health Research Institutes, 35 Keyan Road, Zhunan, Miaoli County 35053, Taiwan; 6 Department of Health Services Administration, China Medical University, No. 91, Hsueh-Shih Road, Taichung 40402, Taiwan; 7 Department of Family Medicine, Min-Sheng General Hospital, 168 ChingKuo Road, Taoyuan 33044, Taiwan; 8 National Center for Geriatrics and Welfare Research, National Health Research Institutes, No. 95-1, Xuefu Rd., Huwei Township, Yunlin County 632007, Taiwan; 9 Section of Endocrinology and Metabolism, Department of Medicine, Taipei Veterans General Hospital, No. 201, Sec. 2, Shipai Road, Beitou District, Taipei 11217, Taiwan; 10 Faculty of Medicine, National Yang Ming Chiao Tung University School of Medicine, Taipei 11221, Taiwan; 11 Department of Medical Education and Research, Kaohsiung Veterans General Hospital, Kaohsiung 81346, Taiwan; 12 Department of Internal Medicine, Taitung Hospital, Ministry of Health and Welfare, Taitung 95043, Taiwan; 13 Master Program in Biomedicine, College of Science and Engineering, National Taitung University, Taitung 95092, Taiwan; 14 Department of Emergency Medicine, Kaohsiung Veterans General Hospital, Kaohsiung 81346, Taiwan; 15 Department of Recreation and Sports Management, Tajen University, Pintung 90741, Taiwan; 16 Department of Urology, College of Medicine and Shu-Tien Urological Research Center, National Yang Ming Chiao Tung University, Taipei 11221, Taiwan; 17 Department of Surgery, Kaohsiung Veterans General Hospital, Kaohsiung 81346, Taiwan

**Keywords:** mirabegron, overactive bladder, antimuscarinic medications, obesity-related neoplasms, site-specific neoplasms, all-cause mortality

## Abstract

**Background/Aims**: Mirabegron, a selective β3-adrenergic receptor agonist used to treat overactive bladder (OAB), can promote adipose tissue browning and alter systemic metabolism, raising interest in its potential relationship with cancer development. However, clinical evidence regarding cancer risk remains limited and inconsistent. We compared the risks of obesity-related and site-specific cancers between patients with OAB initiating mirabegron and those initiating antimuscarinic medications. **Methods**: We conducted a multicenter emulated target trial using the TriNetX Global Collaborative Network. Adults aged ≥20 years with OAB or related symptoms who newly initiated mirabegron or an antimuscarinic medication were included using a new-user, active-comparator design. Patients with pre-existing malignancy were excluded. Propensity score matching was performed 1:1, and a 1-year lag period after treatment initiation was applied for cancer outcomes to reduce potential reverse causation from occult malignancy. Cox proportional hazards models estimated hazard ratios (HRs) and 95% confidence intervals (CIs) under an intention-to-treat analog. **Results**: A 365-day lag/landmark period was applied before propensity-score matching. Patients with a recorded neoplasm or death during this period, or without follow-up extending through day 366, were excluded. Subsequent 1:1 propensity-score matching yielded 106,500 patients in each treatment group. Mirabegron initiation was not associated with the composite of obesity-related neoplasms compared with antimuscarinic initiation (HR, 1.011; 95% CI, 0.954–1.071). The individual neoplasm outcomes also showed no significant between-group differences. However, among patients eligible for the 1-year landmark mortality analysis, mirabegron initiation was associated with higher subsequent all-cause mortality (HR, 1.083; 95% CI, 1.044–1.124). Exploratory negative-control analysis of Hodgkin lymphoma showed no significant association. **Conclusions**: Among patients with OAB, mirabegron initiation was not associated with a significant difference in overall obesity-related neoplasm risk compared with antimuscarinic initiation. The observed associations with all-cause mortality should be interpreted cautiously given the observational design, multiple comparisons, and potential residual confounding. These findings are hypothesis-generating and require confirmation in prospective studies.

## Simple Summary

Mirabegron and antimuscarinic medications are commonly used to treat overactive bladder, but whether mirabegron is associated with cancer risk remains uncertain. We used a large international electronic health record network to compare obesity-related and site-specific neoplasms between patients newly treated with mirabegron and those treated with antimuscarinic medications. After applying a 1-year landmark period and matching patients on baseline characteristics, 106,500 patients were included in each treatment group. We found no significant difference in the overall risk of obesity-related neoplasms or individual neoplasm outcomes between the two groups. An exploratory negative-control analysis of Hodgkin lymphoma also showed no significant association. However, mirabegron use was associated with a modestly higher risk of subsequent all-cause mortality among patients who survived, remained free of neoplasms, and continued to be observed through the 1-year landmark period. Because this was an observational study, these findings cannot establish causality and require confirmation in prospective studies.

## 1. Introduction

Cancer is one of the foremost public health challenges of the 21st century and a major obstacle to further improvements in global life expectancy. In 2021, it accounted for approximately one in six deaths worldwide (17%) and nearly one-quarter of all deaths attributable to noncommunicable diseases (23%). Beyond its substantial health burden, cancer also imposes considerable societal and macroeconomic costs [1,2]. According to the latest GLOBOCAN 2024 estimates, approximately 20.6 million new cancer cases and 9.8 million cancer-related deaths occurred globally in 2024 [1]. Lung cancer remains the most frequently diagnosed malignancy and the leading cause of cancer-related death, followed by cancers of the female breast, colorectum, prostate, and stomach [1,2]. Primarily because of population growth and aging, the annual number of new cancer cases is projected to reach approximately 34.4 million by 2050, with the greatest increases expected in low- and middle-income countries [1,2]. These projections underscore the urgent need to strengthen cancer prevention, early detection, and treatment strategies worldwide [1,2,3].

In contrast to the traditional view that cancer is driven primarily by dysregulated signaling pathways, metabolic reprogramming is now recognized as a defining hallmark of malignancy [4,5]. Cancer cells extensively alter their metabolic processes to obtain the nutrients, energy, biosynthetic intermediates, and growth signals required for sustained proliferation, invasion, and metastasis [5]. One prominent example is their preferential reliance on aerobic glycolysis rather than mitochondrial oxidative phosphorylation, characterized by increased glucose uptake and lactate production [6]. Accumulating evidence suggests that activating brown adipose tissue (BAT) and inducing the browning of white adipose tissue (WAT) can increase energy expenditure and improve metabolic disorders such as type 2 diabetes [7]. Adipose tissue browning refers to the phenotypic transformation of white adipocytes into brown-like thermogenic cells with greater mitochondrial abundance and activity. This process is mediated largely by sympathetic stimulation of β3-adrenergic receptors (β3-ARs), which can be activated by cold exposure or pharmacological agonists [8]. Activated brown and beige adipocytes exhibit enhanced glucose uptake and increased expression of thermogenic markers, particularly uncoupling protein 1 (UCP1). Located in the inner mitochondrial membrane, UCP1 promotes non-shivering thermogenesis by dissipating the mitochondrial proton gradient and releasing energy as heat [9].

Mirabegron, a selective β3-AR agonist, is widely prescribed for the treatment of overactive bladder (OAB) [10]. A meta-analysis of six studies suggested that mirabegron may increase BAT activity, resting energy expenditure, circulating non-esterified fatty acid concentrations, and body temperature. However, it may also elevate heart rate, blood pressure, and circulating insulin levels [11]. Given the close relationship between cellular metabolism and tumor progression, mirabegron-induced adipose tissue browning could plausibly affect cancer development. Nevertheless, preclinical findings have been inconsistent [12,13]. Wei et al. demonstrated that the browning of perirenal adipose tissue promoted the growth, invasion, and metastasis of clear cell renal cell carcinoma, whereas inhibition of adipocyte browning enhanced antitumor efficacy [12]. Conversely, Sun et al. reported that mirabegron suppressed tumor growth and prolonged survival in several experimental tumor models by inducing UCP1-dependent adipose tissue browning and redirecting glucose uptake from tumors to thermogenic adipose tissue [13]. Clinical evidence regarding the potential carcinogenic effects of mirabegron also remains inconclusive [14,15,16,17]. During randomized clinical development programs, neoplasms were reported more frequently among mirabegron recipients than among participants receiving anticholinergic agents or placebo [14,15]. Nevertheless, a post-authorization safety study using propensity score–matched cohorts from Danish, Swedish, UK, and US databases found no evidence of an increased overall cancer risk among mirabegron initiators compared with antimuscarinic initiators [16]. A subsequent nationwide Korean cohort study, however, reported a higher risk of kidney cancer associated with β3-AR agonists than with anticholinergic therapy [17]. Thus, important uncertainty remains regarding specific neoplasm outcomes beyond those emphasized in previous studies. The present study was designed to complement, rather than replicate, this evidence by evaluating a prespecified composite of obesity-related neoplasms and a broader range of site-specific neoplasm outcomes using an international electronic health record (HER)-based data source. We therefore compared these outcomes between new initiators of mirabegron and antimuscarinic medications among patients with OAB.

## 2. Patients and Methods

### 2.1. Study Design and Data Source

We conducted an emulated target trial using data from the TriNetX Global Collaborative Network, a large international platform that integrates real-world EHR data for clinical and health services research. The network contains de-identified longitudinal health information from more than 250 million individuals treated across over 186 participating healthcare organizations worldwide. Before incorporation into the network, data from each participating institution undergo local processing, standardization, and harmonization to facilitate consistent analyses across healthcare systems. Available information includes patient demographics, diagnoses, procedures, medication use, laboratory measurements, and vital signs. The inclusion of both inpatient and outpatient encounters enables longitudinal assessment of clinical exposures and outcomes across different healthcare settings [18]. TriNetX applies systematic data-quality procedures to evaluate data conformance, completeness, and clinical plausibility [19].

Treatment initiation was assessed from 1 January 2013 through 31 December 2025. Patients were followed using all data available after cohort entry until the outcome of interest, recorded death, or the last recorded clinical fact, as applicable. The TriNetX output did not distinguish specific reasons for the end of follow-up. Calendar year, healthcare organization, and geographic region were not available as covariates in the standard analytic module and were therefore not included in propensity-score matching.

### 2.2. Ethics Statement

The TriNetX platform operates in accordance with applicable data privacy and protection requirements, including the Health Insurance Portability and Accountability Act (HIPAA) and the General Data Protection Regulation (GDPR). Because the database provides access exclusively to de-identified patient-level information and all analytical outputs are presented in aggregate form, the Western Institutional Review Board determined that formal ethical review was not required. The study protocol was also reviewed and approved by the Institutional Review Board of Kaohsiung Veterans General Hospital (approval number: KSVGH24-CT3-09). Individual informed consent was waived because this secondary analysis used only de-identified data and aggregate analytical outputs.

### 2.3. Emulated Target Trial Design

The study was conducted and reported in accordance with the TARGET (Transparent Reporting of Observational Studies Emulating a Target Trial) Statement [20] to enhance transparency and methodological rigor. Using an open-label target trial emulation framework, we compared treatment strategies of mirabegron and antimuscarinic medications among patients with OAB. The major components of the hypothetical target trial—including eligibility criteria, treatment strategies, treatment assignment, time zero, follow-up, outcomes, causal estimand, and statistical analysis plan—were defined a priori and then emulated using data from the TriNetX Global Collaborative Network (Supplementary Table S1).

### 2.4. Study Eligibility Criteria

Eligible patients were aged 20 years or older and had overactive bladder or a related lower urinary tract diagnosis recorded within the 365 days before treatment initiation. Eligible diagnoses were urge incontinence (ICD-10-CM N39.41), overactive bladder (N32.81), urgency of urination (R39.15), urinary frequency or polyuria (R35), or other specified urinary incontinence (N39.4) (Supplementary Table S2). TriNetX provided aggregate cohort counts after application of the programmed eligibility and exclusion criteria but did not provide mutually exclusive counts for each individual exclusion criterion. Because individual patients could satisfy multiple exclusion criteria, criterion-specific exclusion counts could not be reliably derived from the aggregate output.

Patients were excluded if, on or before treatment initiation, they had undergone bladder excision, had end-stage renal disease or dialysis dependence, or had a recorded transplanted-organ or tissue status. To establish the post-lag cancer-risk cohort, patients with any recorded neoplasm (ICD-10-CM C00-D49) or recorded death before or during the first 365 days after treatment initiation were excluded. These lag/landmark eligibility criteria were applied before propensity-score matching. Patients who remained eligible at day 366 were subsequently entered into the propensity-score matching procedure.

### 2.5. Treatment Assignment, Index Date, and Follow-Up

We used a new-user, active-comparator design. The mirabegron cohort comprised patients whose first qualifying treatment was mirabegron, whereas the antimuscarinic cohort comprised those whose first qualifying treatment was oxybutynin, darifenacin, trospium, solifenacin, tolterodine, fesoterodine, or propiverine. The treatment-initiation date (exposure index date) was defined as the date of the first qualifying medication record. New use required a 1-year-and-1-day medication-free look-back period with no recorded use of mirabegron or any included antimuscarinic medication. Prior use of either treatment class during this period precluded cohort entry, thereby ensuring mutually exclusive treatment strategies at initiation. Patients were analyzed according to their initial treatment assignment regardless of subsequent discontinuation, addition, or switching, consistent with an intention-to-treat analog. Because the TriNetX medication domain includes electronic medication orders and other medication records contributed by participating healthcare organizations but does not uniformly confirm pharmacy dispensing or medication ingestion, we use the term “medication record” rather than “prescription” or “dispensing.” TriNetX does not require a fixed minimum duration of observable medical history beyond the prespecified look-back period; therefore, remote or incompletely captured prior OAB medication use cannot be entirely excluded.

We distinguished treatment initiation from the analytic time origin for cancer outcomes. A 365-day lag period after treatment initiation was applied to reduce potential reverse causation and the influence of occult malignancy, with cancer outcome assessment beginning on day 366. For Kaplan–Meier and Cox analyses, elapsed follow-up time was reset to day 0 at the end of the lag period; thus, 0 years on the cancer-free survival curves corresponds to day 366 after treatment initiation. Patients with any recorded neoplasm (ICD-10-CM C00–D49) or death during the first 365 days, as well as those without follow-up extending through the lag period, were excluded from the post-lag cancer analyses. This approach also prevented neoplasms diagnosed during the first year from subsequently being counted as incident events because of repeated diagnosis codes. Follow-up duration for cancer outcomes was therefore calculated from the post-lag analytic time zero and excluded the preceding 365-day lag period.

The all-cause mortality analysis used the same 365-day landmark period but was defined separately from the cancer analyses. Patients who died, developed any recorded neoplasm (ICD-10-CM C00–D49), or did not remain under observation through the first 365 days after treatment initiation were excluded from the mortality cohort. Analytic time zero for all-cause mortality was therefore day 366, and subsequent deaths were ascertained from that date through the last available follow-up. Thus, the mortality analysis estimated subsequent mortality among patients who survived, remained neoplasm-free, and were under observation through the 1-year landmark period rather than mortality from treatment initiation among all eligible new users.

After the applicable analytic time zero, patients were followed using all available data until the outcome of interest, recorded death, or the last recorded clinical fact, as appropriate. The TriNetX aggregate output did not distinguish specific reasons for the end of follow-up, such as transfer, disenrollment, administrative end of observation, or true loss to follow-up. Calendar year of treatment initiation, healthcare organization, and geographic region were not available as covariates in the standard analytic module and therefore could not be included in propensity score matching. In the cancer analyses, formal competing-risk cumulative-incidence estimation was unavailable in the standard TriNetX module; therefore, death was treated as a censoring event, and Kaplan–Meier curves were interpreted as cancer-free survival probabilities rather than cumulative incidence functions.

### 2.6. Study Outcomes

The primary outcome was a composite of prespecified obesity-related neoplasms [21]. Secondary outcomes included the individual components of the composite and all-cause mortality. Site-specific outcomes included colorectal cancer (ICD-10-CM C18–C20), breast cancer (C50), malignant neoplasm of the uterine corpus (C54), ovarian cancer (C56), renal cell carcinoma excluding the renal pelvis (C64), pancreatic cancer (C25), hepatocellular carcinoma (C22.0), gallbladder and biliary tract cancer (C23, C24), gastric cancer (C16), esophageal cancer (C15), multiple myeloma (C90.0), and benign cerebral meningioma (D32.0) (Supplementary Table S3). Because the composite included benign cerebral meningioma, the term “obesity-related neoplasms” was used rather than “obesity-related cancers.”

Hodgkin lymphoma (ICD-10-CM C81) and testicular cancer (C62) were evaluated separately as exploratory negative-control outcomes using the same eligibility criteria, 365-day landmark, propensity-score matching specification, matched-cohort framework, and full-follow-up window as the primary analysis. TriNetX suppresses outcome counts below 10 to protect patient privacy; when the event count was suppressed in either cohort, the exact count and comparative effect estimate were not reported.

### 2.7. Covariates and Propensity Score Matching

Baseline covariates were assessed during the 365 days before or on the treatment-initiation date. Prespecified covariates included age, sex, race, body mass index, smoking- and alcohol-related diagnoses, metabolic and cardiovascular diseases, diabetes, renal and hepatic diseases, respiratory diseases, gastroesophageal reflux disease, Barrett esophagus, cholelithiasis, cancer-screening indicators, baseline medications, and available laboratory measurements, including body mass index (BMI) and hemoglobin A1C (HbA1c). To improve control for baseline cognitive vulnerability, frailty-related characteristics, and healthcare utilization, the propensity-score model additionally included cognitive impairment (ICD-10-CM G31.84, R41.3, R41.81, and R41.89), dementia (G30.x, F01.x, F02.x, F03.x, and G31.83), prior inpatient encounters, and emergency-department use. These variables were considered available proxies for frailty and healthcare utilization rather than comprehensive measures of multidimensional frailty (Supplementary Table S4).

TriNetX identifies the number of patients with recorded laboratory or anthropometric measurements but does not provide patient-level data for external imputation or independent reconstruction of the propensity-score model. BMI and HbA1c measurements were incomplete and were not externally imputed. In the principal matched cohort, BMI was recorded for 68,041 of 106,500 mirabegron initiators (63.9%) and 67,114 of 106,500 antimuscarinic initiators (63.0%); HbA1c was recorded for 34,916 (32.8%) and 33,614 (31.6%), respectively. Propensity-score matching was performed within the standard TriNetX Analytics module using the prespecified covariates. However, the aggregate analytic output did not provide sufficient information to determine whether missing continuous measurements were internally handled by exclusion, missing-value indicators or categories, or another platform-specific procedure. Therefore, we could not independently characterize the platform’s internal missing-data handling for propensity-score estimation. The reported means and SMDs for BMI and HbA1c were based on participants with recorded measurements and should not be interpreted as demonstrating balance in these variables across the entire matched cohort.

Propensity-score matching was performed after application of the 365-day lag/landmark eligibility criteria. Thus, the matched cohorts represented patients who were alive, remained free of recorded neoplasms, and had follow-up extending through day 366 after treatment initiation. Propensity scores representing the probability of initiating mirabegron were estimated by logistic regression using all prespecified baseline characteristics. Mirabegron initiators were matched 1:1 to antimuscarinic initiators using nearest-neighbor matching without replacement and a caliper of 0.1 pooled standard deviations. Covariate balance after matching was evaluated using standardized mean differences (SMD), with an absolute standardized mean difference below 0.10 indicating acceptable balance. All cognitive-impairment, dementia, inpatient-use, and emergency-department-use variables met this criterion after matching.

The same cohort logic, covariate set, matching approach, and analysis settings were retained across these runs. Subgroup analyses were constructed and rematched separately within each subgroup using the same propensity-score specification.

### 2.8. Analysis Plan and Statistical Methods

The primary analyses followed an intention-to-treat (ITT) analog, whereby participants were analyzed according to the treatment initiated at the index date, irrespective of subsequent discontinuation, switching, or addition of the comparator medication. Such post-index treatment changes did not lead to treatment-group reassignment or treatment-related censoring. Accordingly, the estimated effects reflected the association with initiation of the assigned treatment strategy rather than the effect of continuous adherence to that therapy.

Continuous variables were summarized as means and standard deviations or medians and interquartile ranges, as available, and categorical variables as counts and percentages. Following propensity-score matching, treatment-only Cox proportional hazards models were fitted, with antimuscarinic initiation as the reference group; no additional covariate adjustment was performed after matching. HRs and 95% CIs were obtained from the standard TriNetX analytic output. The platform provided model-based confidence intervals but did not support matched-pair-stratified Cox models, cluster-robust standard errors, or healthcare-organization-level clustering in this workflow. The proportional hazards assumption was evaluated using the diagnostic test provided by TriNetX, and the resulting diagnostic *p*-values were reported separately from association *p*-values (Supplementary Table S7).

Kaplan–Meier methods were used to estimate overall survival or cancer-free survival, and treatment groups were compared using log-rank tests. Kaplan–Meier estimates for cancer outcomes were not interpreted as cumulative-incidence functions. Because death may preclude a subsequent cancer diagnosis, a competing-risk cumulative-incidence analysis would have been informative; however, Fine–Gray regression could not be implemented within the standard TriNetX Analytics module, and individual-level event-time data were unavailable for external competing-risk modeling. Death was therefore treated as censoring in the cancer analyses. Accordingly, the Cox estimates were interpreted as cause-specific time-to-event associations among individuals remaining alive and under observation, rather than as estimates of the absolute cumulative probability of cancer in the presence of competing death.

All tests were two-sided. To account for multiple comparisons across the 12 site-specific neoplasm outcomes, nominal log-rank *p*-values were adjusted using the Benjamini–Hochberg false discovery rate (BH-FDR) procedure [22], with the 12 outcomes treated as a single family of secondary hypotheses. An FDR-adjusted *p*-value < 0.05 was considered statistically significant. The primary composite outcome, negative-control outcomes, all-cause mortality, subgroup analyses, and targeted sensitivity analyses were not included in this multiplicity-adjustment family and were interpreted separately.

To address differences in follow-up duration between the treatment groups and assess whether treatment associations varied across follow-up periods, we repeated the analyses of the primary composite outcome and all-cause mortality using common 3-year and 5-year follow-up horizons and compared these estimates with those from the full-follow-up analysis. For cancer outcomes, the common-horizon analyses began at the post-lag analytic time zero (day 366 after treatment initiation). Because the proportional hazards assumption may not hold over extended follow-up, particularly for the primary composite outcome, Cox HRs were interpreted as summary associations over the specified follow-up periods when the proportional hazards diagnostic indicated a violation, rather than as constant relative hazards over time. A formal time-varying coefficient analysis was not available from the aggregate analytic output used in this study. To assess the robustness of the gastric cancer findings to a longer latency period, we conducted a sensitivity analysis using a 730-day lag. Patients with any recorded neoplasm or death before or during this lag period were excluded, cancer follow-up began on day 731 after treatment initiation, and the cohorts were rematched using the same covariates and matching procedure. To assess whether the primary findings depended on the inclusion of incompletely recorded BMI and HbA1c values in the propensity-score model, we repeated 1:1 propensity-score matching after excluding these two variables. Diagnoses of type 2 diabetes and overweight/obesity and all other prespecified covariates were retained. Covariate balance was assessed using SMDs, with values <0.10 considered acceptable. Outcome analyses otherwise followed the same 365-day landmark design, follow-up framework, and post-match Cox modeling approach as the primary analysis.

Exploratory subgroup analyses were conducted according to age, sex, race, diabetes status, metabolic dysfunction-associated steatotic liver disease or steatohepatitis (MASLD/MASH; formerly nonalcoholic fatty liver disease/nonalcoholic steatohepatitis [NAFLD/NASH]), BMI, and HbA1c. Each subgroup was constructed and propensity-score matched separately using the same propensity-score specification rather than being selected from the original matched cohort. No imputation of missing BMI or HbA1c values was performed. Because formal interaction tests were unavailable, subgroup findings were considered exploratory, and neither nominal subgroup *p*-values nor differences in statistical significance across subgroups were interpreted as evidence of effect modification.

## 3. Results

### 3.1. Study Population and Baseline Characteristics

**Figure 1:**
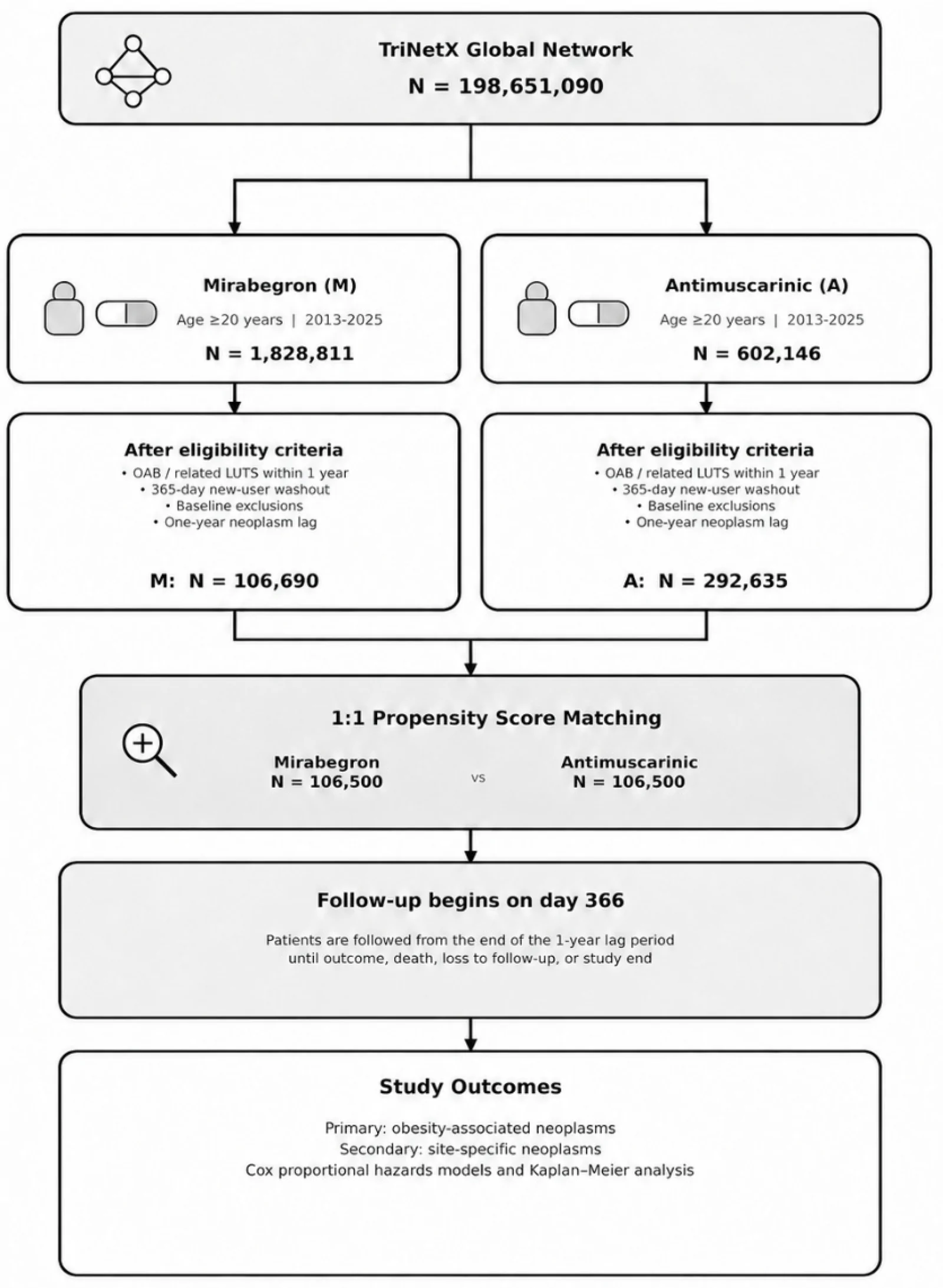
Study flow diagram. The diagram shows cohort construction from the TriNetX Global Collaborative Network through application of the eligibility and exclusion criteria, including the 365-day lag/landmark eligibility criteria, followed by identification of the treatment-specific cohorts and 1:1 propensity-score matching. Patients with a recorded neoplasm or death during the first 365 days after treatment initiation, or without follow-up extending through day 366, were excluded before matching. The resulting matched cohorts entered cancer and mortality follow-up on day 366. TriNetX did not provide mutually exclusive counts for individual exclusion criteria because patients could satisfy more than one criterion; therefore, criterion-specific exclusion counts are not presented. OAB, overactive bladder; LUTS, lower urinary tract symptoms.

After application of the eligibility criteria, including exclusion of patients with a recorded neoplasm or death during the 365-day lag/landmark period and those without follow-up extending through day 366, 106,690 mirabegron users and 292,635 antimuscarinic users were eligible for matching. Before matching, mirabegron users were older (mean age, 63.0 versus 58.7 years) and had a lower mean BMI (30.1 versus 31.1 kg/m^2^). Subsequent 1:1 propensity-score matching yielded 106,500 patients in each group (Table 1 and Figure 1). These matched cohorts constituted the populations entering the day-366 cancer and mortality analyses. All reported baseline characteristics with evaluable data had SMDs < 0.10 after matching, indicating adequate balance in the populations actually analyzed. After matching, mean age was 63.0 ± 15.1 years among mirabegron users and 62.6 ± 14.7 years among antimuscarinic users; women accounted for 72.5% and 73.1%, respectively. Mean BMI was 30.2 ± 7.4 and 30.4 ± 7.5 kg/m^2^, respectively. All reported baseline characteristics with evaluable data had standardized mean differences below 0.1, indicating balance in measured demographics, comorbidities, medications, and cancer-screening indicators (Table 1). The additionally included proxies for cognitive vulnerability, frailty-related characteristics, and healthcare utilization were also well balanced after matching, with SMDs of <0.001–0.009 for cognitive impairment and dementia variables, 0.024 for prior inpatient use, and 0.027 for emergency-department use.

Mean follow-up was 1229 ± 1002 days among mirabegron users and 1578 ± 1268 days among antimuscarinic users; the corresponding medians were 990 and 1276 days.

### 3.2. Overall Obesity-Related Neoplasms and All-Cause Mortality

**Figure 2:**
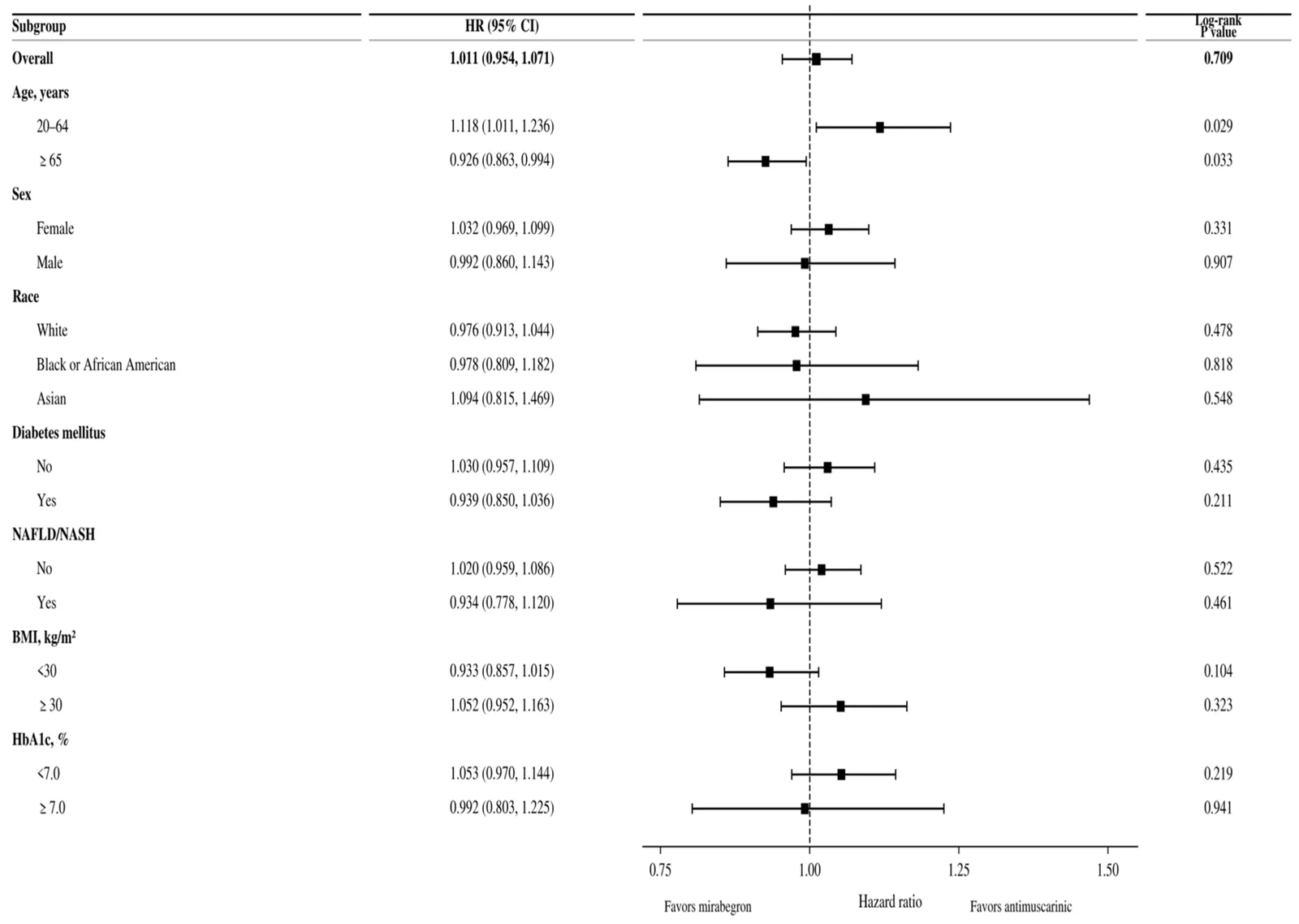
A forest plot presenting the hazard ratios (HRs) and corresponding 95% confidence intervals (CIs) from univariable analyses, with time to the primary outcome of obesity-related neoplasms as the dependent variable. **Notes:** Squares represent the HR estimates, and horizontal bars indicate the corresponding 95% CIs. NAFLD, nonalcoholic fatty liver disease; NASH, nonalcoholic steatohepatitis; BMI, body mass index.

For the composite obesity-related neoplasm outcome, 2046 events occurred among mirabegron initiators and 2803 among antimuscarinic initiators (Table 2). In the full-follow-up analysis, the corresponding HR was 1.011 (95% CI, 0.954–1.071; Figure 2), and the Kaplan–Meier curves showed no significant between-group difference (log-rank *p* = 0.709; Supplementary Figure S1).

Because follow-up duration differed between treatment groups and the proportional hazards assumption was not consistently satisfied over longer follow-up, we gave particular consideration to analyses using common follow-up horizons. The HR for the primary composite outcome was 0.924 (95% CI, 0.859–0.994; log-rank *p* = 0.034) at 3 years, 0.975 (95% CI, 0.915–1.040; *p* = 0.442) at 5 years, and 1.011 (95% CI, 0.954–1.071; *p* = 0.709) over full follow-up (Supplementary Table S5). The proportional hazards diagnostic showed no evidence of violation at 3 years (*p* = 0.696) but indicated violations at 5 years (*p* = 0.002) and over full follow-up (*p* = 0.003). Accordingly, the longer-term Cox HRs should be interpreted as summary associations over their respective follow-up periods rather than as constant relative hazards over time.

**Figure 3:**
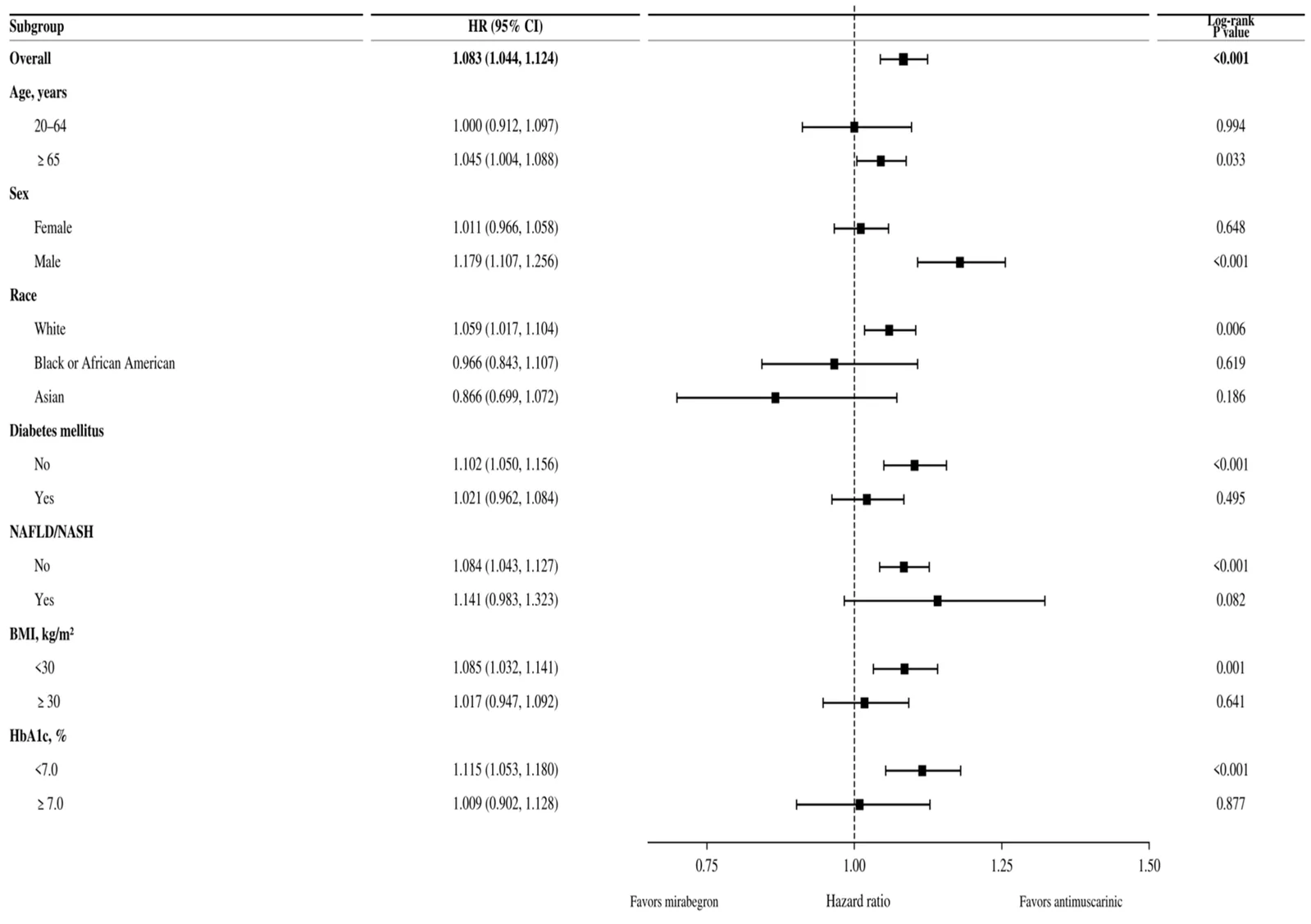
A forest plot presenting the hazard ratios (HRs) and corresponding 95% confidence intervals (CIs) from univariable analyses, with time to all-cause mortality as the dependent variable. **Notes:** Squares represent the HR estimates, and horizontal bars indicate the corresponding 95% CIs. NAFLD, nonalcoholic fatty liver disease; NASH, nonalcoholic steatohepatitis; BMI, body mass index.

In the landmark mortality cohort comprising patients who survived, remained neoplasm-free, and were under observation through the 365-day lag period, mirabegron initiation was associated with a higher subsequent risk of all-cause mortality than antimuscarinic initiation (HR, 1.083; 95% CI, 1.044–1.124; Figure 3). Survival probability was lower among mirabegron users, with a significant difference between the survival curves (log-rank *p* < 0.001; Supplementary Figure S2).

### 3.3. Individual Neoplasm Outcomes

Event counts for the site-specific outcomes are provided in Table 2. No significant differences were observed for colorectal cancer (HR, 1.022; 95% CI, 0.876–1.192), breast cancer (HR, 0.959; 95% CI, 0.878–1.049), endometrial cancer as labeled in the analysis (HR, 0.855; 95% CI, 0.687–1.065), ovarian cancer (HR, 0.887; 95% CI, 0.674–1.167), kidney cancer (HR, 1.190; 95% CI, 0.969–1.462), pancreatic cancer (HR, 0.960; 95% CI, 0.774–1.189), hepatocellular carcinoma (HR, 0.887; 95% CI, 0.666–1.181), gallbladder or biliary tract cancer (HR, 0.991; 95% CI, 0.651–1.509), esophageal cancer (HR, 1.500; 95% CI, 0.972–2.316), multiple myeloma (HR, 1.003; 95% CI, 0.791–1.272), and meningioma (HR, 1.107; 95% CI, 0.896–1.367). All corresponding confidence intervals included unity (Table 2). Proportional hazards diagnostics showed no evidence of violation for most site-specific outcomes; however, potential violations were observed for breast cancer (*p* = 0.004) and multiple myeloma (*p* = 0.002) (Supplementary Table S6). Accordingly, the corresponding Cox hazard ratios should be interpreted cautiously as summary estimates over follow-up.

After adjustment for multiple comparisons using the BH-FDR procedure, none of the 12 site-specific neoplasm outcomes remained statistically significant. In particular, in the updated 1-year-lag analysis, mirabegron initiation was not significantly associated with gastric cancer (HR, 1.130; 95% CI, 0.796–1.604; nominal log-rank *p* = 0.493), and the association remained nonsignificant after BH-FDR adjustment (*p* = 0.740). Accordingly, the site-specific findings should be considered exploratory (Table 2 and Table S7). In the 2-year-lag sensitivity analysis, 100,944 patients were matched in each treatment group, with 40 and 59 gastric cancer events among mirabegron and antimuscarinic initiators, respectively. No significant between-group association was observed (HR, 1.097; 95% CI, 0.728–1.652; log-rank *p* = 0.657), and the proportional hazards diagnostic showed no evidence of violation (*p* = 0.426).

### 3.4. Exploratory Negative-Control Outcomes

In the harmonized negative-control analysis, 102,166 patients were included in each matched cohort. Hodgkin lymphoma occurred in 15 mirabegron users and 20 antimuscarinic users, with no evidence of a between-group difference (HR, 1.019; 95% CI, 0.519–1.999; log-rank *p* = 0.958). The proportional hazards diagnostic showed no evidence of violation (*p* = 0.066). For testicular cancer, the event count was below the TriNetX reporting threshold in at least one cohort; therefore, the exact event count and comparative effect estimate were not reported (Supplementary Table S8).

### 3.5. Sensitivity Analysis Excluding BMI and HbA1c

Before matching, this analysis included 102,324 mirabegron and 291,389 antimuscarinic users; 102,158 patients remained in each cohort after 1:1 matching. All 67 retained covariates had post-match SMDs <0.10. Excluding BMI and HbA1c did not materially alter the findings. The HRs were 0.985 (95% CI, 0.929–1.045; *p* = 0.622) for obesity-related neoplasms, 1.018 (95% CI, 0.712–1.456; *p* = 0.922) for gastric cancer, and 1.127 (95% CI, 1.086–1.170; *p* < 0.001) for all-cause mortality (Supplementary Table S9).

### 3.6. Subgroup Analyses

Subgroup analyses were generally consistent with the primary findings, although several nominally significant associations were observed (Figure 2 and Figure 3). For the composite obesity-related neoplasm outcome, mirabegron initiation was associated with a nominally higher hazard among participants aged 20–64 years (HR, 1.118; 95% CI, 1.011–1.236) and a nominally lower hazard among those aged ≥65 years (HR, 0.926; 95% CI, 0.863–0.994) (Figure 2). No significant associations were observed across the sex, race, diabetes, MASLD/MASH, or BMI subgroups. Because formal interaction tests were unavailable, the contrasting estimates between age groups should not be interpreted as evidence of effect modification. In the separately rematched HbA1c ≥ 7% subgroup, 6750 patients were included in each treatment group. The composite obesity-related neoplasm outcome occurred in 158 mirabegron and 201 antimuscarinic initiators (HR, 0.992; 95% CI, 0.803–1.225; log-rank *p* = 0.941), providing no evidence of a significant between-group association.

For all-cause mortality, higher hazards associated with mirabegron initiation were observed among participants aged ≥65 years, men, White participants, those without diabetes or MASLD/MASH, those with HbA1c < 7%, and participants in BMI < 30 strata (Figure 3). In the separately rematched HbA1c ≥ 7% subgroup, 6750 patients were included in each treatment group, with 548 deaths among mirabegron initiators and 738 among antimuscarinic initiators. However, no significant between-group difference in all-cause mortality was observed in this subgroup (HR, 1.009; 95% CI, 0.902–1.128; log-rank *p* = 0.877).

## 4. Discussion

In this multicenter target trial emulation of individuals with OAB, mirabegron initiation was not associated with a statistically significant difference in the composite obesity-related neoplasm outcome, and none of the individual neoplasm comparisons reached statistical significance after adjustment for multiple comparisons. These null findings should not, however, be interpreted as establishing equivalence or excluding clinically relevant differences for individual outcomes with relatively wide confidence intervals. For the primary composite outcome, the association varied across follow-up horizons: a modest inverse association was observed at 3 years, whereas no significant between-group differences were found at 5 years or over full follow-up. Notably, the proportional hazards assumption was satisfied at 3 years but violated at 5 years and over full follow-up. The longer-term HRs should therefore be interpreted as summary measures over the respective follow-up periods rather than as constant relative hazards over time. These time-horizon findings suggest that the comparative association may vary during follow-up and caution against relying solely on the full-follow-up HR. Given the number of site-specific outcomes evaluated, these analyses should be considered exploratory. After BH-FDR adjustment for multiple comparisons, none of the site-specific neoplasm outcomes remained statistically significant; therefore, isolated nominal associations should not be regarded as confirmatory evidence of treatment-related differences in site-specific neoplasm risk. Similarly, subgroup findings should be considered hypothesis-generating because formal interaction tests were unavailable. In contrast, mirabegron initiation was associated with a higher risk of all-cause mortality.

Activation of BAT thermogenesis and WAT browning may increase competition for glucose between adipose tissue and tumors [13]. Consistent with this mechanism, positron emission tomography–computed tomography (PET–CT) using fluorine-18 fluorodeoxyglucose (^18^F-FDG) demonstrated reduced tumor glucose uptake following mirabegron-induced BAT activation, potentially restricting glycolysis-dependent cancer metabolism [13]. Genetic deletion of UCP1 abolished tumor suppression in mice, suggesting that non-shivering thermogenesis is essential to this response [13], consistent with mirabegron’s predominantly UCP1-dependent metabolic effects [23]. Beyond their metabolic actions, β3-ARs have emerged as potential therapeutic targets in melanoma, breast cancer, and colorectal cancer because of their capacity to modulate the tumor immune microenvironment (TIME) [24,25]. Nevertheless, experimental findings vary. Sun et al. reported suppressed tumor growth in hepatocellular carcinoma and pancreatic ductal adenocarcinoma models [13], whereas Wei et al. found that thermogenic perirenal adipocytes promoted clear cell renal cell carcinoma (ccRCC) progression through adipokine secretion and metabolic remodeling [12]. In murine RCC models, Park et al. observed that mirabegron-induced perirenal adipose browning initially promoted primary tumor progression but subsequently suppressed growth; lung metastatic burden nevertheless increased by more than 41% across the phases examined [26]. These findings suggest that the effects depend on fat depot, cancer type, and immune context [26]. Importantly, direct extrapolation of these preclinical findings to humans should be approached cautiously because substantial species-specific differences exist between rodents and humans, including differences in the relative distribution of β-adrenergic receptor subtypes, intrinsic thermogenic capacity, and the anatomical distribution and metabolic activity of BAT [17].

Clinical evidence is similarly heterogeneous. In a 52-week double-blind trial, malignant, benign, or unspecified neoplasms occurred in 11/820 patients receiving mirabegron 100 mg (1.3%), compared with 1/812 receiving 50 mg (0.1%) and 4/812 receiving tolterodine 4 mg (0.5%) [14]. In one 12-week phase 2/3 trial, serious neoplasm-related adverse events were numerically more frequent with mirabegron 50 mg (3/442; 0.7%) or 100 mg (2/433; 0.5%) than placebo (1/453; 0.2%), without statistical significance [15]. Five other 12-week trials showed no comparable imbalance [27,28,29,30,31]. Our findings should be interpreted in the context of previous comparative safety studies. A propensity score–matched study using Danish, Swedish, UK, and US databases found no increased overall cancer risk among mirabegron initiators compared with antimuscarinic initiators, including analyses of early and subsequent follow-up [16]. Our study extends this evidence by examining a prespecified composite of obesity-related neoplasms and multiple site-specific outcomes in an international EHR-based population. In contrast, a Korean nationwide cohort study reported a higher risk of kidney cancer among β3-AR agonist users than among anticholinergic users [17]. In our analysis, the estimate for renal cell carcinoma was not statistically significant (HR, 1.190; 95% CI, 0.969–1.462); however, the confidence interval includes potentially clinically relevant increases in risk. Accordingly, our findings should not be interpreted as excluding an increased risk of renal cell carcinoma, and further studies with greater outcome-specific precision are warranted.

The gastric cancer findings warrant cautious interpretation. Gastric cancer has heterogeneous etiologies, including pathways associated with reflux and gastric atrophy [32], and obesity is an established risk factor [33]. Although BMI, obesity, gastroesophageal reflux disease, Barrett’s esophagus, and proton pump inhibitor use were balanced after matching, these measures may not fully capture lifetime adiposity, the severity or duration of reflux, or underlying gastric mucosal pathology. Residual confounding related to smoking, geographic variation, endoscopic surveillance, and the classification of gastroesophageal junction tumors also cannot be excluded. Importantly, the updated analyses showed no significant association between mirabegron initiation and gastric cancer in the primary 1-year-lag analysis, after adjustment for multiple comparisons, or in the 2-year-lag sensitivity analysis. Although excluding patients with diagnosed neoplasms before and during the lag period reduced the potential influence of pre-existing disease, occult malignancy cannot be entirely excluded. Previous evidence showing no increase in overall cancer risk [16] further supports caution in interpreting site-specific estimates as evidence of generalized carcinogenicity. Given the limited number of gastric cancer events and the observational design, firm conclusions cannot be drawn, and our findings do not provide robust evidence of an association between mirabegron initiation and gastric cancer.

Medicare data indicate that frail patients are more likely to receive mirabegron after adjustment for age and insurance-related factors, while frailty independently predicts mortality [34]. Preferential prescribing to more vulnerable patients could therefore contribute to the observed mortality association despite adequate balance in recorded comorbidities. Although mirabegron users were older before matching, achieving balance in age does not necessarily ensure comparability in functional status, cognition, physiological reserve, or overall frailty. Medication costs and access barriers may also influence treatment selection [34,35], although whether these factors preferentially selected patients with more severe OAB or poorer underlying health in our cohort remains unknown. Because patients with prior use of either treatment class during the prespecified look-back period were excluded, prior antimuscarinic failure should not be assumed to explain the observed association. Our mortality findings also differ from previous multinational evidence showing no increased risk of cardiovascular or all-cause mortality among mirabegron users [36]. However, direct comparisons are limited by differences in cohort construction and follow-up definitions. In the present study, the mortality analysis used a 365-day landmark design and included only patients who survived, remained free of recorded neoplasms, and continued to be observed throughout the first year after treatment initiation, with mortality follow-up beginning on day 366. The resulting HR therefore reflects subsequent mortality within this selected landmark cohort rather than mortality from treatment initiation among all eligible new users. Although we incorporated available proxies for cognitive vulnerability, frailty-related characteristics, and healthcare utilization—including cognitive impairment, dementia, prior inpatient encounters, and emergency-department use—these measures do not comprehensively capture functional dependence, illness severity, physiological reserve, or multidimensional frailty. Residual confounding by underlying health status may therefore partly explain the observed mortality association. Together, these differences in cohort selection, follow-up definition, and measurement of frailty warrant caution when comparing our mortality findings with those of previous studies [36].

Clinically, the absence of a significant overall obesity-related neoplasm association offers limited reassurance during the observed follow-up but does not establish equivalent long-term safety or support prescribing mirabegron for cancer prevention. The mortality signals alone should not prompt routine discontinuation or preferential antimuscarinic prescribing. Treatment decisions should remain individualized according to symptom burden, response, cognitive vulnerability, cardiovascular status, concomitant medications, affordability, and patient preferences [10]. Additional endoscopic screening solely because of mirabegron exposure is not justified by these findings; evaluation should follow established indications and individual risk profiles. Replication incorporating absolute risk differences, cause-specific mortality, detailed frailty assessment, cancer latency, treatment changes, and early-event exclusions is needed before clinical recommendations change.

Several limitations warrant acknowledgment. First, information on family history, education, geographic factors, and lifestyle characteristics was unavailable. Although the new-user, active-comparator design and alignment of exposure assessment with follow-up were intended to reduce confounding by indication and immortal time bias, residual confounding and selection bias remain possible. Propensity-score matching balanced measured baseline characteristics; however, the standard TriNetX analytic workflow provided model-based confidence intervals and did not support matched-pair-stratified Cox models, cluster-robust standard errors, or healthcare-organization-level clustering. The reported confidence intervals therefore may not fully account for dependence introduced by matching or clustering within healthcare organizations. Moreover, BMI and HbA1c were incompletely recorded, and the exact handling of missing continuous covariates by the platform could not be independently verified for the analytic settings used. However, a sensitivity analysis excluding both variables from propensity-score matching, while retaining diagnoses of diabetes and overweight/obesity, yielded well-balanced cohorts and materially unchanged estimates. This supports the robustness of our principal findings to this modeling uncertainty, although residual confounding from incompletely measured adiposity and glycemic control cannot be excluded. Although a prespecified medication-free look-back period was used to identify new users, TriNetX does not require a fixed minimum duration of observable history beyond this period; consequently, remote or incompletely captured prior use of OAB medications may not have been fully identified. Second, although cognitive impairment, dementia, prior inpatient encounters, and emergency-department use were incorporated as available proxies for frailty and healthcare utilization, TriNetX does not provide comprehensive measures of functional status, dependence, or multidimensional frailty. Residual confounding by underlying health status may therefore persist. Treatment was classified according to the therapy initiated at cohort entry under an intention-to-treat analog, without accounting for subsequent discontinuation, switching, combination therapy, or adherence. Accordingly, the findings should be interpreted as associations with treatment initiation rather than effects of sustained treatment exposure. Third, reliance on diagnostic codes without standardized imaging or pathological confirmation may have resulted in outcome misclassification, the direction of which is uncertain. In addition, medication records could not confirm actual dispensing, medication use, or adherence. Fourth, no untreated OAB comparator was included. Comparing two established pharmacological treatments may improve comparability with respect to treatment indications and clinical settings, while requiring treatment records reduces concerns about identifying OAB solely from diagnostic codes. Nevertheless, the findings reflect relative outcomes between the two treatment strategies and cannot establish their risks compared with no pharmacological treatment. Finally, incomplete ascertainment of mortality and the inability to perform formal competing-risk analyses may have affected the estimates. Fine–Gray regression was unavailable in the standard TriNetX Analytics module, and individual-level event-time data were inaccessible for external modeling. Death was therefore treated as a censoring event in the cancer analyses, and the Cox estimates should be interpreted as relative cause-specific time-to-event associations among individuals who remained alive and under observation rather than as absolute cumulative probabilities of cancer in the presence of competing mortality. Furthermore, the mortality analysis used a 365-day landmark period and excluded patients who died, developed a neoplasm, or were no longer under observation during the first year after treatment initiation. The mortality findings are therefore conditional on survival, absence of neoplasm, and continued observation through this period and should not be interpreted as the effect of treatment initiation on mortality among all eligible new users. Evidence of nonproportional hazards for the primary composite outcome in the 5-year and full-follow-up analyses further limits interpretation of a single constant HR across extended follow-up. Although common-horizon analyses were used to characterize associations over clinically relevant follow-up periods, a formal time-varying coefficient analysis was not available from the aggregate analytic output. Collectively, these limitations preclude causal inference and underscore the need for confirmation in prospective studies.

## 5. Conclusions

In this multicenter target trial emulation involving individuals with OAB, the risks of overall obesity-related neoplasm and any individual obesity-related neoplasm did not differ significantly between mirabegron and antimuscarinic initiators. Mirabegron initiation was, however, associated with a higher risk of all-cause mortality. These observational findings are hypothesis-generating and warrant prospective confirmation before causal conclusions can be drawn.

## Figures and Tables

**Table 1:** Baseline characteristics of mirabegron and antimuscarinic users before and after propensity score matching among patients with overactive bladder.

Characteristic	Before PSM			After PSM ^a^		
	Mirabegron *n* = 106,690	Antimuscarinic *n* = 292,635	SMD	Mirabegron *n* = 106,500	Antimuscarinic *n* = 106,500	SMD
**Demographics**						
Age at index, years	63.0 ± 15.1	58.7 ± 15.7	0.277	63.0 ± 15.1	62.6 ± 14.7	0.026
Gender						
Male	29,335 (27.5)	87,171 (29.8)	0.051	29,306 (27.5)	28,643 (26.9)	0.014
Female	77,355 (72.5)	205,464 (70.2)	0.051	77,194 (72.5)	77,857 (73.1)	0.014
Race						
White	69,498 (65.1)	193,705 (66.2)	0.022	69,383 (65.1)	69,012 (64.8)	0.007
Black or African American	8925 (8.4)	34,766 (11.9)	0.117	8921 (8.4)	8753 (8.2)	0.006
Asian	5128 (4.8)	14,314 (4.9)	0.004	5125 (4.8)	4869 (4.6)	0.011
**Diagnoses**						
Type 2 diabetes mellitus	18,553 (17.4)	53,585 (18.3)	0.024	18,516 (17.4)	17,744 (16.7)	0.019
Hypertensive diseases	41,793 (39.2)	121,171 (41.4)	0.046	41,722 (39.2)	40,000 (37.6)	0.033
Disorders of lipoprotein metabolism and other lipidemias	39,703 (37.2)	108,647 (37.1)	0.002	39,605 (37.2)	37,903 (35.6)	0.033
Overweight and obesity	19,601 (18.4)	61,470 (21.0)	0.066	19,567 (18.4)	18,677 (17.5)	0.022
Fatty liver, not elsewhere classified	3441 (3.2)	9880 (3.4)	0.008	3431 (3.2)	3215 (3.0)	0.012
Nonalcoholic steatohepatitis	449 (0.4)	1126 (0.4)	0.006	448 (0.4)	442 (0.4)	0.001
Chronic hepatitis B with delta agent	12 (0.0)	32 (0.0)	<0.001	12 (0.0)	10 (0.0)	0.002
Chronic hepatitis B without delta agent	345 (0.3)	911 (0.3)	0.002	345 (0.3)	325 (0.3)	0.003
Chronic hepatitis C	547 (0.5)	2433 (0.8)	0.039	546 (0.5)	476 (0.4)	0.010
Fibrosis and cirrhosis of liver	754 (0.7)	2303 (0.8)	0.009	753 (0.7)	680 (0.6)	0.008
Chronic kidney disease	7344 (6.9)	19,213 (6.6)	0.013	7320 (6.9)	7028 (6.6)	0.011
Gastroesophageal reflux disease	26,940 (25.3)	73,820 (25.2)	0.001	26,862 (25.2)	25,727 (24.2)	0.025
Barrett’s esophagus	958 (0.9)	2218 (0.8)	0.015	950 (0.9)	902 (0.8)	0.005
Cholelithiasis	3053 (2.9)	8812 (3.0)	0.009	3048 (2.9)	2911 (2.7)	0.008
Chronic obstructive pulmonary disease	6040 (5.7)	18,581 (6.3)	0.029	6030 (5.7)	5651 (5.3)	0.016
Asthma	10,340 (9.7)	30,809 (10.5)	0.028	10,316 (9.7)	9806 (9.2)	0.016
Obstructive sleep apnea	10,488 (9.8)	28,414 (9.7)	0.004	10,442 (9.8)	9959 (9.4)	0.015
Ischemic heart diseases	12,721 (11.9)	33,085 (11.3)	0.019	12,685 (11.9)	12,194 (11.4)	0.014
Heart failure	4772 (4.5)	12,943 (4.4)	0.002	4762 (4.5)	4569 (4.3)	0.009
Cerebrovascular diseases	9275 (8.7)	22,939 (7.8)	0.031	9223 (8.7)	8905 (8.4)	0.011
Colorectal cancer screening encounter	13,202 (12.4)	35,201 (12.0)	0.011	13,171 (12.4)	12,702 (11.9)	0.013
Screening mammography encounter	20,084 (18.8)	53,196 (18.2)	0.017	20,039 (18.8)	19,503 (18.3)	0.013
Cervical cancer screening encounter	7810 (7.3)	23,222 (7.9)	0.023	7805 (7.3)	7676 (7.2)	0.005
Mild cognitive impairment	2091 (2.0)	3134 (1.1)	0.073	2004 (1.9)	1924 (1.8)	0.006
Other amnesia	5170 (4.8)	10,398 (3.6)	0.064	5077 (4.8)	4880 (4.6)	0.009
Age-related cognitive decline	103 (0.1)	159 (0.1)	0.015	99 (0.1)	92 (0.1)	0.002
Other cognitive symptoms and signs	2013 (1.9)	3477 (1.2)	0.057	1949 (1.8)	1865 (1.8)	0.006
Alzheimer’s disease	1223 (1.1)	1542 (0.5)	0.068	1154 (1.1)	1123 (1.1)	0.003
Vascular dementia	626 (0.6)	1102 (0.4)	0.030	607 (0.6)	600 (0.6)	0.001
Dementia in other diseases	1822 (1.7)	2300 (0.8)	0.083	1723 (1.6)	1641 (1.5)	0.006
Unspecified dementia	2648 (2.5)	4365 (1.5)	0.071	2578 (2.4)	2515 (2.4)	0.004
Neurocognitive disorder with Lewy bodies	247 (0.2)	257 (0.1)	0.036	226 (0.2)	206 (0.2)	0.004
**Procedures**						
Diagnostic colonoscopy	3294 (3.1)	9377 (3.2)	0.007	3282 (3.1)	3179 (3.0)	0.006
Colonoscopy with foreign-body removal	35 (0.0)	81 (0.0)	0.003	34 (0.0)	28 (0.0)	0.003
Colonoscopy with biopsy	1900 (1.8)	5728 (2.0)	0.013	1895 (1.8)	1821 (1.7)	0.005
Colonoscopy with submucosal injection	58 (0.1)	207 (0.1)	0.007	57 (0.1)	56 (0.1)	<0.001
Colonoscopy with control of bleeding	51 (0.0)	114 (0.0)	0.004	50 (0.0)	42 (0.0)	0.004
Colonoscopy with hot biopsy forceps removal	84 (0.1)	242 (0.1)	0.001	83 (0.1)	84 (0.1)	<0.001
Colonoscopy with snare removal	678 (0.6)	2153 (0.7)	0.012	675 (0.6)	636 (0.6)	0.005
Endoscopic inspection of lower intestinal tract	1303 (1.2)	4201 (1.4)	0.019	1299 (1.2)	1264 (1.2)	0.003
Screening mammography	15,193 (14.2)	31,404 (10.7)	0.106	15,105 (14.2)	14,832 (13.9)	0.007
**Medications**						
Metformin	10,880 (10.2)	30,310 (10.4)	0.005	10,849 (10.2)	10,376 (9.7)	0.015
Insulins and analogues	8751 (8.2)	25,436 (8.7)	0.018	8726 (8.2)	8330 (7.8)	0.014
GLP-1 receptor agonists	4029 (3.8)	8565 (2.9)	0.047	4003 (3.8)	3909 (3.7)	0.005
SGLT2 inhibitors	2985 (2.8)	5949 (2.0)	0.050	2956 (2.8)	2909 (2.7)	0.003
DPP-4 inhibitors	2862 (2.7)	6429 (2.2)	0.031	2847 (2.7)	2723 (2.6)	0.007
Thiazolidinediones	1101 (1.0)	3303 (1.1)	0.009	1100 (1.0)	1070 (1.0)	0.003
HMG-CoA reductase inhibitors	30,600 (28.7)	76,238 (26.1)	0.059	30,476 (28.6)	29,333 (27.5)	0.024
Fibrates	2140 (2.0)	6176 (2.1)	0.007	2135 (2.0)	1979 (1.9)	0.011
Aspirin	19,924 (18.7)	51,430 (17.6)	0.029	19,844 (18.6)	19,018 (17.9)	0.020
Nonsteroidal anti-inflammatory drugs	43,544 (40.8)	120,210 (41.1)	0.005	43,436 (40.8)	42,223 (39.6)	0.023
Proton pump inhibitors	29,691 (27.8)	77,007 (26.3)	0.034	29,577 (27.8)	28,521 (26.8)	0.022
ACE inhibitors	13,836 (13.0)	44,327 (15.1)	0.063	13,824 (13.0)	13,120 (12.3)	0.020
Angiotensin II receptor blockers	15,215 (14.3)	35,928 (12.3)	0.058	15,145 (14.2)	14,504 (13.6)	0.017
Beta-blocking agents	26,217 (24.6)	68,224 (23.3)	0.030	26,124 (24.5)	25,176 (23.6)	0.021
Selective calcium-channel blockers	15,998 (15.0)	41,589 (14.2)	0.022	15,946 (15.0)	15,311 (14.4)	0.017
Diuretics	21,731 (20.4)	60,943 (20.8)	0.011	21,676 (20.4)	20,758 (19.5)	0.022
Estrogens	16,016 (15.0)	33,499 (11.4)	0.105	15,921 (14.9)	15,793 (14.8)	0.003
Systemic hormonal contraceptives	6631 (6.2)	21,973 (7.5)	0.051	6620 (6.2)	6473 (6.1)	0.006
**Laboratory measurements**						
Body mass index, kg/m^2^	30.1 ± 7.4	31.1 ± 8.0	0.128	30.2 ± 7.4	30.4 ± 7.5	0.037
Hemoglobin A1C, %	6.1 ± 1.2	6.2 ± 1.4	0.057	6.1 ± 1.2	6.1 ± 1.3	0.052
**Healthcare utilization**						
Emergency department services	24,146 (22.6)	76,991 (26.3)	0.086	24,091 (22.6)	22,912 (21.5)	0.027
Inpatient encounter	26,834 (25.2)	78,890 (27.0)	0.041	26,768 (25.1)	25,655 (24.1)	0.024

Data are presented as mean ± standard deviation or *n* (%). PSM, propensity score matching; SMD, standardized mean difference; GLP-1, glucagon-like peptide-1; SGLT2, sodium–glucose cotransporter 2; DPP-4, dipeptidyl peptidase-4; ACE, Angiotensin-converting enzyme. Race categories shown in the table do not encompass the entire cohort; remaining patients were recorded as other or unknown or had missing race information. Percentages were calculated using the full cohort as the denominator, and missing race data were not imputed. BMI and HbA1c were incompletely recorded and were not externally imputed. After matching, BMI was available for 68,041 (63.9%) mirabegron and 67,114 (63.0%) antimuscarinic initiators; HbA1c was available for 34,916 (32.8%) and 33,614 (31.6%), respectively. The reported means and SMDs for BMI and HbA1c were calculated among patients with recorded measurements and therefore do not establish balance in these variables across the entire matched cohort. Propensity-score matching was performed within the standard TriNetX Analytics module; its aggregate output did not provide sufficient information to independently determine how missing continuous measurements were handled internally during propensity-score estimation. ^a^ Propensity score matching was performed using the prespecified demographic, clinical, procedural, medication, laboratory, and healthcare-utilization variables listed in the table.

**Table 2:** Hazard ratios for neoplasm outcomes comparing mirabegron with antimuscarinic therapy.

Outcomes	Mirabegron Events	Antimuscarinic Events	HR (95%CI) *	*p*-Value
Obesity-related neoplasms	2046	2803	1.011 (0.954–1.071)	0.709
Colorectal cancer	290	387	1.022 (0.876–1.192)	0.782
Breast cancer	846	1206	0.959 (0.878–1.049)	0.361
Endometrial cancer	134	211	0.855 (0.687–1.065)	0.161
Ovary cancer	86	132	0.887 (0.674–1.167)	0.391
Kidney cancer	174	200	1.190 (0.969–1.462)	0.097
Pancreas cancer	143	213	0.960 (0.774–1.189)	0.706
Hepatocellular carcinoma	79	121	0.887 (0.666–1.181)	0.411
Gallbladder/biliary tract cancer	38	54	0.991 (0.651–1.509)	0.967
Gastric cancer	58	73	1.130 (0.796–1.604)	0.493
Esophageal	43	43	1.500 (0.972–2.316)	0.065
Multiple myeloma	119	169	1.003 (0.791–1.272)	0.981
Meningioma	156	209	1.107 (0.896–1.367)	0.345
All-cause mortality	5059	6816	1.083 (1.044–1.124)	<0.001

HR, hazard ratio; CI, confidence interval. *: Hazard ratios were estimated using Cox proportional hazards regression in the propensity score–matched cohort after balancing measured baseline confounders.

## Data Availability

Data are not available for data protection reasons. It can only be obtained upon reasonable request. Further information about the data of TriNetX can be accessed on their website: https://trinetx.com/?mc_cid=7e2ecd5bc5&mc_eid=%5BUNIQID%5D.

## Supplementary Materials

The following supporting information can be downloaded at https://www.mdpi.com/article/10.3390/cancers18203279/s1, Supplementary Figure S1: Kaplan–Meier curves showing cancer-free survival probability for obesity-related neoplasm among propensity score–matched mirabegron and antimuscarinic initiators; Supplementary Figure S2: Overall survival probability among mirabegron and antimuscarinic users; Supplementary Table S1: Specification and emulation of the target trial comparing mirabegron with antimuscarinic therapy for the risk of obesity-associated neoplasms using the TriNetX global collaborative Network; Supplementary Table S2: Relevant codes used for inclusion and exclusion criteria; Supplementary Table S3: Relevant codes used for study outcomes; Supplementary Table S4: Relevant codes used for covariates; Supplementary Table S5: Common-horizon sensitivity analyses comparing 3-year, 5-year, and full follow-up outcomes between mirabegron and antimuscarinic users; Supplementary Table S6: Proportional hazards assumption diagnostics for study outcomes among mirabegron and antimuscarinic users; Supplementary Table S7: Benjamini–Hochberg false discovery rate analysis of outcomes between mirabegron and antimuscarinic users; Supplementary Table S8: Harmonized exploratory negative-control analyses among mirabegron and antimuscarinic users; Supplementary Table S9: Primary and sensitivity analyses excluding BMI and HbA1c from propensity-score matching in mirabegron versus antimuscarinic users.

## Abbreviations

The following abbreviations are used in this manuscript: β3-AR, β3-adrenergic receptor; BAT, brown adipose tissue; BMI, body mass index; CI, confidence interval; EHR, electronic health record; HbA1c, glycated hemoglobin; HR, hazard ratio; MASLD, metabolic dysfunction-associated steatotic liver disease; MASH, metabolic dysfunction-associated steatohepatitis; NAFLD, nonalcoholic fatty liver disease; NASH, nonalcoholic steatohepatitis; OAB, overactive bladder; PSM, propensity score matching; SMD, standardized mean difference; TIME, tumor immune microenvironment; UCP1, uncoupling protein 1; WAT, white adipose tissue.
