# Supplementary material for: Comparative Neoplasm Risk of Mirabegron and Antimuscarinic Therapy in Overactive Bladder: A Multicenter Target Trial Emulation
Source: Cancers (Basel). 2026 Oct 12;18(20):3279. doi: 10.3390/cancers18203279 (PMC13702379; doi:10.3390/cancers18203279)
Supplement: Supplementary file 1 [file cancers-18-03279-s001.zip › cancers-4571539-supplementary.pdf]

Supplementary Materials

Supplementary Table S1. Specification and emulation of the target trial comparing mirabegron with antimuscarinic therapy for the risk of obesity-associated neoplasms using the TriNetX global collaborative Network.

**Protocol**	**Specification of Target Trials**	**Emulation of Target Trials**
**Eligibility criteria**	● Adults aged ≥20 years during the study period from January 1, 2013, through December 31, 2025.<br>● A documented diagnosis of overactive bladder (OAB) or related lower urinary tract symptoms.<br>● No history before the index date of malignancy, cystectomy, pelvic radiotherapy, bladder cancer–directed treatment, end-stage renal disease or dialysis, or solid organ transplantation.	The definitions and procedures were consistent with those prespecified in the target trial framework. New use required a 1-year-and-1-day medication-free look-back period with no recorded mirabegron or included antimuscarinic medication. Prior use of either class during this period precluded cohort entry. No additional fixed minimum duration of observable history was required beyond this prespecified look-

		back period.
**Treatment strategies**	Initiation of mirabegron or an antimuscarinic medication after satisfying the prespecified new-user and medication-free look-back criteria.	Participants were classified according to the first qualifying medication record. The treatment-initiation date was defined as the date of the first qualifying mirabegron or antimuscarinic medication record. Treatment strategies were mutually exclusive at treatment initiation.
**Treatment assignment**	At treatment initiation, eligible participants were assigned to either mirabegron or antimuscarinic therapy	Participants were classified according to the treatment initiated at cohort entry and analyzed according to this initial treatment assignment,

		irrespective of subsequent discontinuation, addition, or switching, consistent with an intention-to-treat analog.
**Index date / analytic time zero**	For both cancer and mortality outcomes, a 365-day lag period was applied following treatment initiation to minimize potential reverse causation and the influence of occult malignancy. The analytic time zero for both cancer and mortality outcomes was defined as day 366 after treatment initiation.	The treatment-initiation date was distinguished from the analytic time origin for cancer and mortality outcomes. After the 365-day lag period, elapsed follow-up time was reset to day 0 on treatment-initiation day 366. Patients with a recorded neoplasm or death before or during the lag period were excluded from the post-lag cancer analyses.
**Outcomes**	The primary outcome was a composite of obesity- related neoplasms. Secondary outcomes included each individual	The definitions and procedures were implemented in accordance with those prespecified in the target trial

	component of the obesity- related neoplasm composite and all-cause mortality.	framework.
**Follow-up**	For both cancer and mortality outcomes, follow-up began after completion of the 365-day lag period and continued until the occurrence of the outcome of interest or the end of available follow-up. Accordingly, both analyses evaluated subsequent outcomes among patients who survived, remained free of recorded neoplasms, and continued to be observed throughout the 365-day landmark period.	Cancer and mortality follow-up began at analytic time zero (day 366 after treatment initiation) and continued until the first occurrence of the outcome of interest or the last recorded clinical fact. Patients without follow-up extending through the lag period did not contribute to post-lag cancer follow-up.
**Causal contrast of interest**	Intention-to-treat approach	Under the intention-to-treat–analog approach.

**Statistical analysis**	Risks of study outcomes were compared between treatment strategies using Cox proportional hazards regression models. Kaplan–Meier methods were used to estimate event-free probabilities during follow-up, and between-group differences were assessed using the log-rank test.	The analysis followed the prespecified target trial framework. The 365-day lag/landmark eligibility criteria were applied before propensity-score matching. Propensity scores were then estimated among patients remaining eligible at day 366 using the prespecified baseline covariates, followed by 1:1 nearest-neighbor matching. After matching, treatment-only Cox proportional hazards models were fitted with antimuscarinic initiation as the reference; no additional covariate adjustment was performed.

Supplementary Table S2. Relevant codes used for inclusion and exclusion criteria.

Category	Condition / Definition	Coding System	Codes
Inclusion	Overactive bladder	ICD-10-CM	N32.81
Inclusion	Urge incontinence	ICD-10-CM	N39.41
Inclusion	Urinary urgency	ICD-10-CM	R39.15
Inclusion	Urinary frequency or polyuria	ICD-10-CM	R35
Inclusion	Other specified urinary incontinence	ICD-10-CM	N39.4
Inclusion	Age ≥20 years	Demographic	Age at index date
Exposure	Mirabegron	ATC	G04BD12
Active comparator	Oxybutynin	ATC	G04BD04
Active comparator	Tolterodine	ATC	G04BD07
Active comparator	Solifenacin	ATC	G04BD08
Active comparator	Trospium	ATC	G04BD09
Active comparator	Darifenacin	ATC	G04BD10
Active comparator	Fesoterodine	ATC	G04BD11

Exclusion	Any malignant neoplasm before index	ICD-10-CM	C00–C96
Exclusion	End-stage renal disease	ICD-10-CM	N18.6
Exclusion	Dependence on renal dialysis	ICD-10-CM	Z99.2
Exclusion	Encounter for dialysis	ICD-10-CM	Z49.*
Exclusion	Solid organ transplant status	ICD-10-CM	Z94.*
Exclusion	Cystectomy	Procedure	TriNetX procedure concept/code set
Exclusion	Pelvic radiotherapy	Procedure	TriNetX procedure concept/code set
Exclusion	Bladder cancer–directed treatment	Diagnosis/Procedure/Medication	TriNetX concept/code set

Note: OAB, overactive bladder; ICD-10-CM, International Classification of Diseases, Tenth Revision, Clinical Modification; ATC, Anatomical Therapeutic Chemical classification system. Procedure-based exclusions were identified using the corresponding standardized concepts available within the TriNetX Analytics Platform.

Supplementary Table S3. Relevant codes used for study outcomes.

Outcome	Definition	Coding System	Codes
Primary	Composite obesity-associated neoplasms	ICD-10-CM	Composite of the cancer outcomes listed below
Secondary	Colorectal cancer	ICD-10-CM	C18–C20
Secondary	Breast cancer	ICD-10-CM	C50
Secondary	Malignant neoplasm of uterine corpus	ICD-10-CM	C54
Secondary	Ovarian cancer	ICD-10-CM	C56
Secondary	Kidney cancer, excluding renal pelvis	ICD-10-CM	C64
Secondary	Pancreatic cancer	ICD-10-CM	C25
Secondary	Hepatocellular carcinoma	ICD-10-CM	C22.0
Secondary	Gallbladder cancer	ICD-10-CM	C23
Secondary	Other/unspecified biliary tract cancer	ICD-10-CM	C24
Secondary	Gastric cancer	ICD-10-CM	C16
Secondary	Esophageal cancer	ICD-10-CM	C15
Secondary	Multiple myeloma	ICD-10-CM	C90.0

Secondary	Benign cerebral meningioma	ICD-10-CM	D32.0
Secondary	All-cause mortality	EHR	Death record
Negative control	Hodgkin lymphoma	ICD-10-CM	C81
Negative control	Malignant neoplasm of testis	ICD-10-CM	C62

Note: EHR, electronic health record; ICD-10-CM, International Classification of Diseases, Tenth Revision, Clinical Modification. Hodgkin lymphoma and testicular cancer were prespecified exploratory negative-control outcomes and were analyzed separately; neither was included in the primary obesity-related neoplasm composite. For these outcomes, the same eligibility criteria, 365-day landmark, propensity-score matching specification, and full-follow-up framework as the primary analysis were applied. Benign cerebral meningioma (D32.0) was included as a prespecified component of the obesity-related neoplasm composite. Patients with any recorded neoplasm (C00–D49), including pre-existing meningioma, before or during the 365-day lag period were excluded from the post-lag cancer analyses.

Supplementary Table S4. Relevant codes used for covariates.

Covariate	Coding System	Codes
Age	Demographic	Age at index date
Sex	Demographic	Male/Female
Race	Demographic	White; Black or African American; Asian; other/unknown
Body mass index	LOINC	39156-5
Hypertensive diseases	ICD-10-CM	I10–I15
Disorders of lipoprotein metabolism	ICD-10-CM	E78.*
Gastroesophageal reflux disease	ICD-10-CM	K21.*
Overweight and obesity	ICD-10-CM	E66.*
Type 2 diabetes mellitus	ICD-10-CM	E11.*
Ischemic heart diseases	ICD-10-CM	I20–I25
Obstructive sleep apnea	ICD-10-CM	G47.33
Cerebrovascular diseases	ICD-10-CM	I60–I69
Asthma	ICD-10-CM	J45.*
Chronic kidney disease	ICD-10-CM	N18.*
Chronic obstructive pulmonary disease	ICD-10-CM	J44.*
Heart failure	ICD-10-CM	I50.*
Fatty liver	ICD-10-CM	K76.0
Cholelithiasis	ICD-10-CM	K80.*
Barrett esophagus	ICD-10-CM	K22.7*
Fibrosis and cirrhosis of liver	ICD-10-CM	K74.*

Chronic hepatitis C	ICD-10-CM	B18.2
Chronic hepatitis B without delta-agent	ICD-10-CM	B18.1
Nonalcoholic steatohepatitis	ICD-10-CM	K75.81
Chronic hepatitis B with delta-agent	ICD-10-CM	B18.0
Cognitive impairment	ICD-10-CM	G31.84, R41.3, R41.81, R41.89
Dementia	ICD-10-CM	G30.x, F01.x, F02.x, F03.x, G31.83
Screening for malignant neoplasm of colon	ICD-10-CM	Z12.11
Screening for malignant neoplasm of breast	ICD-10-CM	Z12.31, Z12.39
Screening for malignant neoplasm of cervix	ICD-10-CM	Z12.4
NSAIDs	ATC	M01A*
Proton pump inhibitors	ATC	A02BC*
HMG-CoA reductase inhibitors (statins)	ATC	C10AA*
Beta-blocking agents	ATC	C07*
Diuretics	ATC	C03*
Aspirin	ATC	B01AC06
Selective calcium channel blockers	ATC	C08*
Angiotensin II receptor blockers	ATC	C09C*
ACE inhibitors	ATC	C09A*
Estrogens	ATC	G03C*
Metformin	ATC	A10BA02
Insulins and analogues	ATC	A10A*
Hormonal contraceptives	ATC	G03A*

Hemoglobin A1C	LOINC	4548-4

Note: ACE, angiotensin-converting enzyme; ATC, Anatomical Therapeutic Chemical classification system; BMI, body mass index; HbA1c, glycated hemoglobin; ICD-10-CM, International Classification of Diseases, Tenth Revision, Clinical Modification; LOINC, Logical Observation Identifiers Names and Codes; NSAIDs, nonsteroidal anti-inflammatory drugs. Baseline covariates were assessed during the 12 months preceding or on the index date.

Supplementary Table S5. Common-horizon sensitivity analyses comparing 3-year, 5-year, and full follow-up outcomes between mirabegron and antimuscarinic users.

Outcome	Follow-up window	Mirabegron events	Antimuscarinic events	HR (95% CI)	Log-rank p value	PH diagnostic p value
**Obesity-related neoplasms**	3-year follow-up	1,300	1,603	0.924 (0.859-0.994)	0.034	0.696
	5-year follow-up	1,710	2,116	0.975 (0.915-1.040)	0.442	0.002
	Full follow-up	2,046	2,803	1.011 (0.954-1.071)	0.709	0.003
**All-cause mortality**	3-year follow-up	2,910	3,161	1.060 (1.008-1.115)	0.023	0.446
	5-year follow-up	4,061	4,715	1.065 (1.021-1.111)	0.003	0.780
	Full follow-up	5,059	6,816	1.083 (1.044-1.124)	<0.001	0.570

Note: HRs compare mirabegron with antimuscarinic therapy. A 365-day lag/landmark period was applied to all analyses in this table. For the mortality analysis, patients who died, developed a recorded neoplasm, or did not remain under observation through the first 365 days after treatment initiation were excluded; mortality follow-up began on day 366. Thus, the mortality estimates represent subsequent mortality among patients who survived, remained neoplasm-free, and were under observation through the 1-year landmark period rather than mortality from treatment initiation among all eligible new users. The 3-year window extended from day 366 through day 1460 after treatment initiation, and the 5-year window extended through day 2190; full follow-up had no prespecified end date. Log-rank p values assess between-group survival differences. PH diagnostic p values test the proportional hazards assumption and were not used as association p values.

Supplementary Table S6. Proportional hazards assumption diagnostics for study outcomes among mirabegron and antimuscarinic users.

Outcome	PH diagnostic p	Interpretation
Colorectal cancer	0.313	No evidence of violation
Breast cancer	0.004	Potential violation
Endometrial cancer	0.350	No evidence of violation
Ovarian cancer	0.778	No evidence of violation
Renal cell carcinoma	0.734	No evidence of violation
Pancreatic cancer	0.064	No evidence of violation
Hepatocellular carcinoma	0.625	No evidence of violation
Gallbladder or biliary tract cancer	0.641	No evidence of violation
Gastric cancer	0.785	No evidence of violation
Esophageal cancer	0.583	No evidence of violation
Multiple myeloma	0.002	Potential violation
Meningioma	0.996	No evidence of violation

Note: PH, proportional hazards. A diagnostic p value <0.05 indicates evidence against the proportional hazards assumption, whereas a p value ≥0.05 indicates no evidence of violation. These diagnostic p values were used solely to assess the proportional hazards assumption and were not interpreted as tests of treatment-outcome associations.

Supplementary Table S7. Benjamini–Hochberg false discovery rate analysis of outcomes between mirabegron and antimuscarinic users.

Outcome	Mirabegron events	Antimuscarinic events	HR (95% CI)	Raw p	BH-FDR p	FDR <0.05
Colorectal cancer	290	387	1.022 (0.876-1.192)	0.782	0.938	No
Breast cancer	846	1,206	0.959 (0.878-1.049)	0.361	0.705	No
Endometrial cancer	134	211	0.855 (0.687-1.065)	0.161	0.644	No
Ovarian cancer	86	132	0.887 (0.674-1.167)	0.391	0.705	No
Renal cell carcinoma	174	200	1.190 (0.969-1.462)	0.097	0.582	No
Pancreatic cancer	143	213	0.960 (0.774-1.189)	0.706	0.938	No
Hepatocellular carcinoma	79	121	0.887 (0.666-1.181)	0.411	0.705	No
Gallbladder or biliary tract cancer	38	54	0.991 (0.651-1.509)	0.967	0.981	No
Gastric cancer	58	73	1.130 (0.796-	0.493	0.740	No

			1.604)			
Esophageal cancer	43	43	1.500 (0.972-2.316)	0.065	0.582	No
Multiple myeloma	119	169	1.003 (0.791-1.272)	0.981	0.981	No
Meningioma	156	209	1.107 (0.896-1.367)	0.345	0.705	No

HR, hazard ratio; CI, confidence interval; BH-FDR, Benjamini-Hochberg false discovery rate. Event counts denote patients with the outcome. Raw p values are association p values from the log-rank tests.

Supplementary Table S8. Harmonized exploratory negative-control analyses among mirabegron and antimuscarinic users.

Outcome	Matched N (M/A)	Events (M/A)	HR (95% CI)	Log-rank P	PH-test P
Hodgkin lymphoma	102,166 / 102,166	15 / 20	1.019 (0.519–1.999)	0.958	0.066
Testicular cancer	102,166 / 102,166	Suppressed	Not reportable	Not reportable	Not reportable

Note: M, mirabegron; A, antimuscarinic; HR, hazard ratio; CI, confidence interval; PH, proportional hazards. Hodgkin lymphoma and testicular cancer were analyzed separately using the same eligibility criteria, 365-day landmark, propensity-score matching specification, matched-cohort framework, and full-follow-up window as the primary analysis. The exact testicular cancer event count was suppressed by TriNetX because fewer than 10 events occurred in at least one cohort; therefore, the HR, 95% CI, and P values were not reported. Suppressed counts were not treated as zero or reconstructed.

Supplementary Table S9. Primary and sensitivity analyses excluding BMI and HbA1c from propensity-score matching in mirabegron versus antimuscarinic users.

**Outcome**	**Analysis**	**Events M / A**	**HR (95% CI)**	**Log-rank P**	**PH-test P**
Obesity-related neoplasms	Primary	2,046 / 2,803	1.011 (0.954–1.071)	0.709	0.003
	Sensitivity	1,967 / 2,742	0.985 (0.929–1.045)	0.622	<0.001
Gastric cancer	Primary	58 / 73	1.130 (0.796–1.604)	0.493	0.785
	Sensitivity	54 / 73	1.018 (0.712–1.456)	0.922	0.060
All-cause mortality	Primary	5,059 / 6,816	1.083 (1.044–1.124)	<0.001	0.570
	Sensitivity	5,038 / 6,499	1.127 (1.086–1.170)	<0.001	0.995

Note: A, antimuscarinic; M, mirabegron; HR, hazard ratio; PH, proportional hazards. The sensitivity analysis excluded BMI and HbA1c from the propensity-score matching specification while retaining diagnoses of type 2 diabetes mellitus and overweight/obesity and all other prespecified covariates. HRs compare mirabegron with antimuscarinic therapy, with antimuscarinic therapy as the reference group. A 365-day lag/landmark period was applied, and outcome follow-up began on day 366 after treatment initiation. Log-rank P values assess between-group

differences, whereas PH-test P values assess the proportional hazards assumption and should not be interpreted as tests of treatment–outcome associations. P values are two-sided.

Supplementary Figure Legends

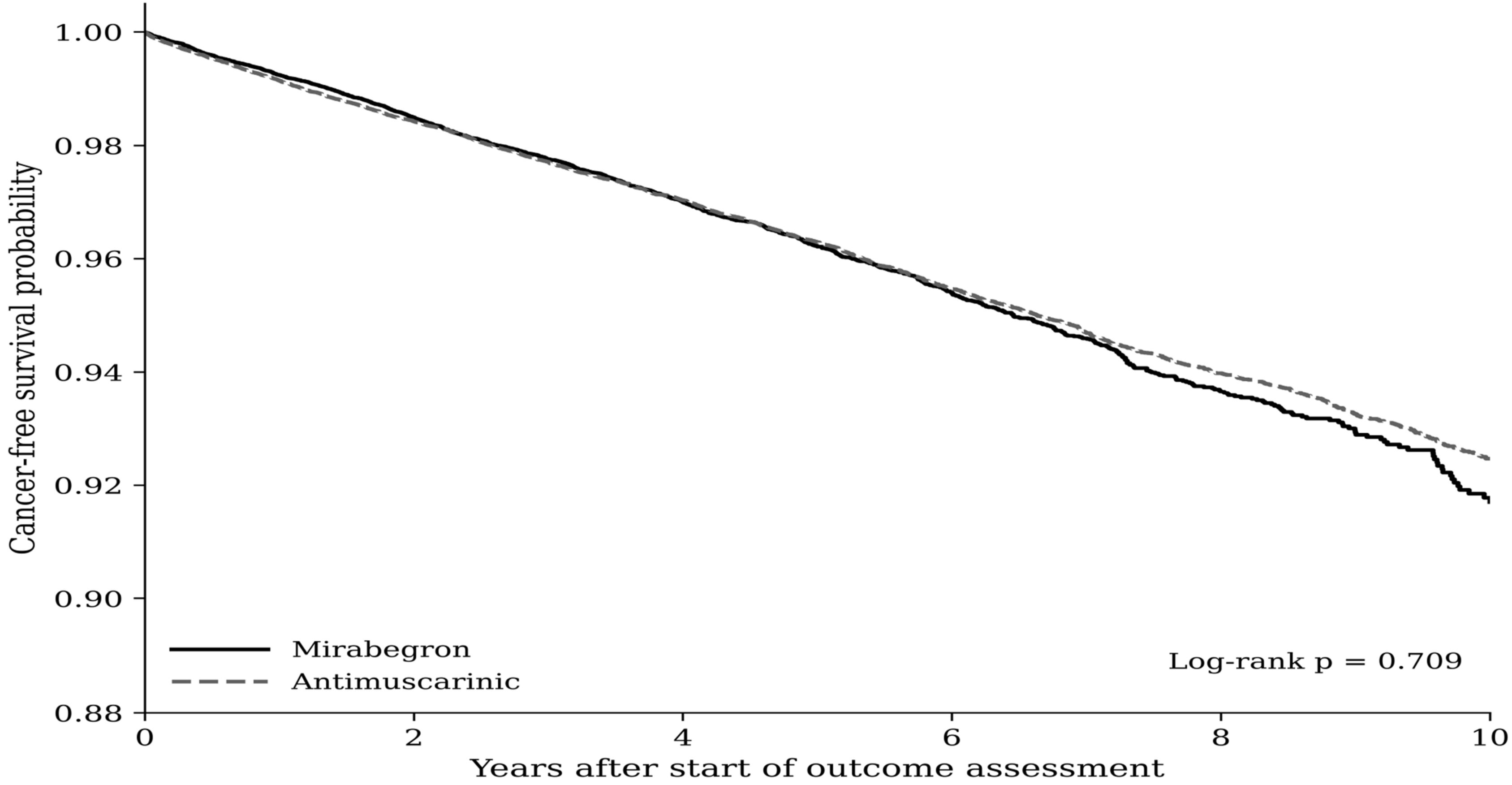

Supplementary Figure S1. Kaplan–Meier curves showing cancer-free survival probability for obesity-related neoplasm among propensity score–matched mirabegron and antimuscarinic initiators.

Note: For cancer outcomes, a 365-day lag period was applied after treatment initiation, and the analytic time axis was reset at the end of the lag period. Accordingly, 0 years on the x-axis corresponds to day 366 after treatment initiation. Patients with a recorded neoplasm or death during the lag period, as well as those without follow-up extending through the lag period, were excluded from the post-lag cancer analysis. The y-axis represents cancer-free survival probability and should not be interpreted as a competing-risk cumulative-incidence function. Actual numbers at risk at post-lag time zero and subsequent time points were not available from the aggregate TriNetX output and therefore are not presented.

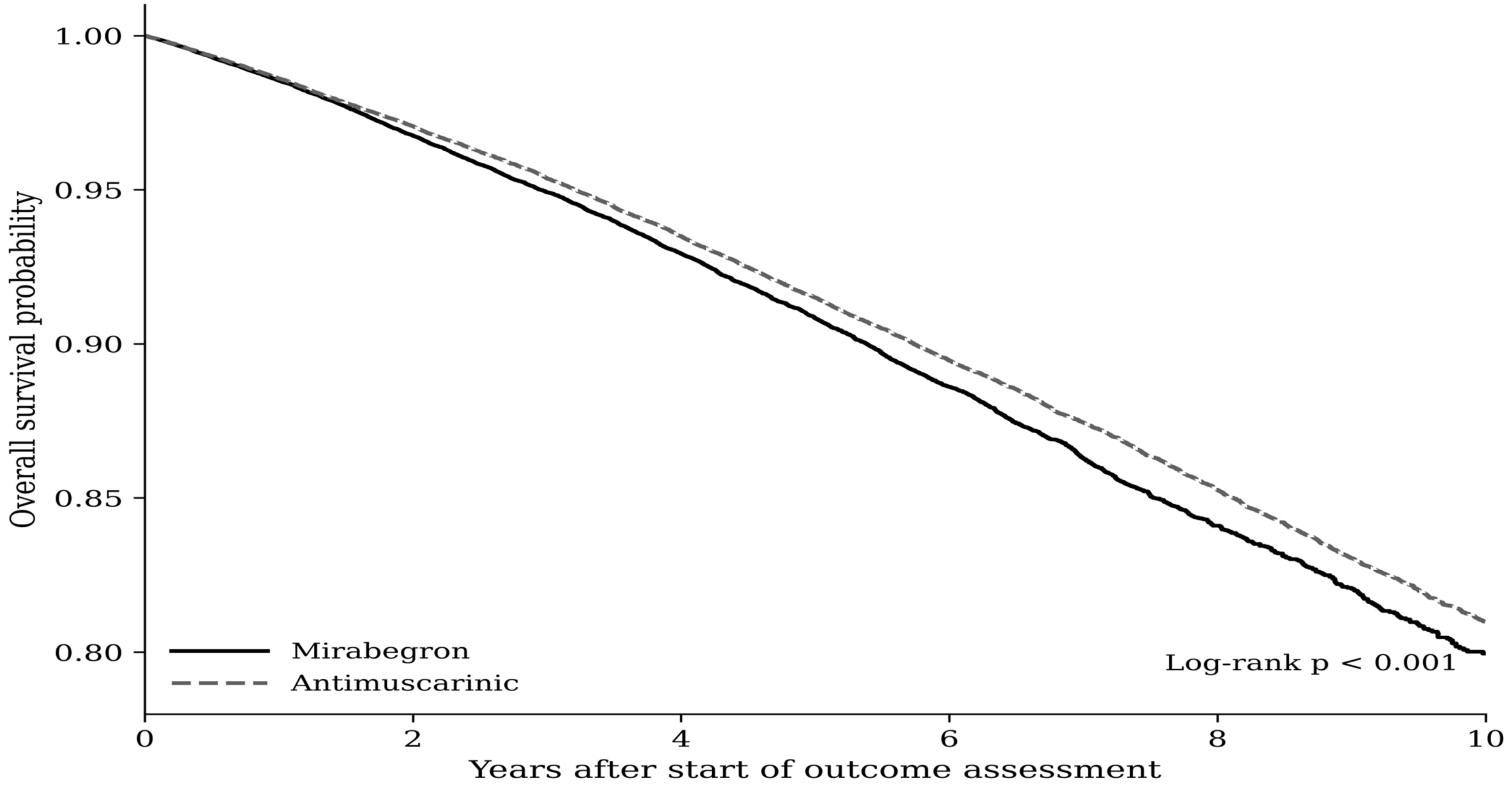

Supplementary Figure S2. Overall survival probability among mirabegron and antimuscarinic users.

Note: A 365-day landmark period was applied to the all-cause mortality analysis. Patients who died, developed a recorded neoplasm, or did not

remain under observation during the first 365 days after treatment initiation were excluded. Mortality follow-up began on day 366 after treatment initiation; accordingly, 0 years on the x-axis corresponds to day 366. The survival curves therefore represent subsequent overall survival among patients who survived, remained neoplasm-free, and were under observation through the 1-year landmark period rather than survival from treatment initiation among all eligible new users. Actual numbers at risk at the landmark time zero and subsequent time points were not available from the aggregate TriNetX output and therefore are not presented.
